# Association of S100B Serum Level With Postoperative Cognitive Dysfunction (POCD) in Non‐Cardiac Surgery: A Meta‐Analysis Study

**DOI:** 10.1002/hsr2.72400

**Published:** 2026-04-16

**Authors:** Saba Asefi, Pouya Omidi, Kimia Jazi, Azadeh Fateh, Dorsa Bahrami Zanjanbar, Simin Sadeghi, Mohammadreza Hajiesmaeili

**Affiliations:** ^1^ School of Medicine Iran University of Medical Sciences Tehran Iran; ^2^ Department of Neurosurgery, School of Medicine Isfahan University of Medical Sciences Isfahan Iran; ^3^ School of Medicine Shahid Beheshti University of Medical Sciences Tehran Iran; ^4^ School of Medicine Shahid Sadoughi University of Medical Sciences Yazd Iran; ^5^ Pharmaceutical Science Research Center, Tehran Medical Sciences Islamic Azad University Tehran Iran; ^6^ GI Pharmacology Interest Group (GPIG) Universal Scientific Education and Research Network (USERN) Tehran Iran; ^7^ Department of Pharmacoeconomics and Pharma Management, School of Pharmacy Shahid Beheshti University of Medical Sciences Tehran Iran; ^8^ Critical Care Quality Improvement Research Center, Loghman Hakim Hospital Shahid Beheshti University of Medical Sciences Tehran Iran

**Keywords:** Cognitive dysfunction, POCD, Postoperative cognitive dysfunction, S100B, surgery

## Abstract

**Background and Aims:**

Postoperative cognitive dysfunction (POCD) is defined as a persistent decline in cognitive performance after a surgical procedure, for which no cause can be identified. S100B is a calcium‐binding protein present in many organs, including the brain. Serum levels have been suggested to be related to POCD. The purpose of this systematic review and meta‐analysis is to assess the association between S100B serum levels and cognitive dysfunction after non‐cardiac surgeries.

**Methods:**

We conducted a systematic search using PubMed/Medline, Scopus, Web of Science, and Google Scholar databases to identify studies. Studies measuring pre‐ and post‐operative serum S100B levels in patients undergoing non‐cardiac surgeries have been included. A random effects model was applied to determine the association of the levels of S100B with POCD.

**Results:**

Of 280 studies screened, 11 articles were eligible for further analysis. The pooled results for pre‐ and post‐treatment outcomes showed no significant differences. The pooled odds ratios (OR) for POCD after non‐cardiac surgeries were 1.77 (95% CI = 1.17–2.67). Subgroup analysis showed that noticeable differences among age (≥ 65 and < 65 years) and continent (Asia vs. Europe) groups. The higher OR observed in the ≥ 65 years' group (OR = 1.94; 95% CI = 0.98‐3.84), and studies from Europe (OR = 1.85; 95% CI = 0.79–4.33).

**Conclusion:**

Overall, current evidence shows that the serum level of S100B is associated with the occurrence of POCD. The S100B monitor may help in the early diagnosis of POCD and the development of preventive strategies.

**Abbreviations**
CNS: Central Nervous System
CRP: C‐Reactive Protein
DSM‐V: Diagnostic and Statistical Manual of Mental Disorders, Fifth Edition
HIPEC: Hyperthermic Intraperitoneal Chemotherapy
ISPOCD: International Study of Postoperative Cognitive Dysfunction
MeSH: Medical Subject Headings
MMSE: Mini‐Mental State Exam
NOS: Newcastle‐Ottawa Quality Assessment Scale
OR: Odds Ratio
POCD: Postoperative Cognitive Dysfunction
PRISMA: Preferred Reporting Items for Systematic Reviews and Meta‐Analyses
RALRP: Robotic‐Assisted Laparoscopic Radical Prostatectomy
S100B: Calcium‐binding protein S100B
SMD: Standardized Mean Difference

## 1 Introduction

Postoperative cognitive dysfunction (POCD), known as the cognitive decline in Diagnostic and Statistical Manual of Mental Disorders, Fifth Edition (DSM‐V), is a mild neurological disorder caused by routine surgery and anesthesia [1]. Post‐surgical decreased memory, cognitive flexibility, language fluency, information processing, social skills, orientation, and attention could lead to a significant increased rates of morbidity and mortality [2, 3, 4, 5]. The condition is more prevalent among patients aged 65 and above who undergo cardiac or orthopedic operations [6, 7]. Consistently, the incidence of POCD was found 1.5 times higher among patients over 60 years in non‐cardiac surgeries [8]. Up to 50% of individuals undergoing hip arthroplasty has shown POCD for a week and in 10%–14% the cognitive decline lasted 3 months after the procedure [9, 10].

Despite decades of research, the exact cause of this cognitive loss is still not fully understood; however, a growing body of experimental and translational studies reported neuroinflammation as a key contributing factor to cerebral damage [11]. POCD considerably lengthen hospital stays, interfere with the healing process following surgery, and decrease the quality of life [12]. This underscores the critical need for developing reliable biomarkers that would enable early diagnosis to enable in time intervention strategies that could mitigate the impact of cognitive decline on recovery, overall morbidity, and mortality.

S100B is a calcium‐binding protein synthesized by Schwann cells and astrocytes, physiologically improving the contact between neural and glial cells [13]. Following CNS injury, glial cells may become activated, resulting in the release of S100B into the bloodstream. Elevated serum levels of S100B may indicate either glial injury or reactive astrogliosis, phenomena that could be associated with either advantageous or harmful outcomes [14]. A previous meta‐analysis confirmed the correlation between S100B levels and POCD [15]. Serum levels of S100B protein may notably rise after cardiac and non‐cardiac surgeries, particularly in the presence of POCD, suggesting an available biomarker [16, 17, 18, 19]; however, the reliability after non‐cardiac surgeries remains vague. Besides handful evaluations on non‐cardiac surgeries, there are multiple confounding factors that could influence the efficacy of S100B as diagnostic biomarker such as age, type of surgery, anesthesia, and geographical area [20, 21]. Importantly, there are also disparities among the peak time of S100B concentrations from minutes to days [15, 22, 23].

Although a previous meta‐analysis by Peng et al. reported an association between peripheral inflammatory markers such as S100B and POCD, that study combined both cardiac and non‐cardiac surgeries, despite the fact that cardiac procedures involve unique sources of neuroinflammation (e.g., cardiopulmonary bypass) that may confound S100B elevations. Moreover, the prior analysis did not provide subgroup evaluations by age or geographical region, factors that may substantially influence postoperative inflammatory responses and cognitive outcomes. Therefore, an evidence gap remains regarding the specific role of S100B in non‐cardiac surgeries and whether demographic or regional factors modify this association. The present study addresses these limitations by focusing exclusively on non‐cardiac surgery populations and conducting detailed subgroup analyses based on age and continent, thereby providing a more precise and context‐specific understanding of the relationship between S100B levels and POCD.

Moreover, cardiac surgeries bring unique challenges like cardiopulmonary bypass, microemboli, reduced blood flow to the brain, and intense inflammation. These factors can raise S100B levels on their own and affect cognitive outcomes, making it hard to interpret the results. To get a clearer picture, our study focuses on non‐cardiac surgeries. This systematic review and meta‐analysis aim to assess the association between POCD after non‐cardiac surgeries and serum S100B levels compared to non‐POCD controls.

## 2 Methods and Materials

This review complies with the Preferred Reporting Items for Systematic Reviews and Meta‐Analyses (PRISMA) standards [24]. This study was approved by the ethics committee of the Shahid Beheshti University of Medical Sciences (IR.SBMU.RETECH.REC.1403.551).

### 2.1 Search Strategy

The present study is a systematic review and meta‐analysis to evaluate the association between S100B levels and POCD in patients undergoing non‐cardiac surgeries compared to controls. The literature search was conducted in January 2025. We searched PubMed/Medline, Scopus, Web of Science, and Google Scholar databases for relevant studies. The search strategy included a combination of MeSH terms and free‐text keywords, such as S100B, S100 beta, postoperative cognitive dysfunction, POCD, surgery, non‐cardiac surgery, and cognitive impairment, using Boolean operators AND/OR as appropriate. Additionally, the reference lists of eligible studies were manually screened to identify further relevant articles. Only publications in English were included.

### 2.2 Inclusion and Exclusion Criteria

We included all the studies that reported the pre‐ and pos‐operation concentrations of S100B and its association with POCD among patients undergone non‐cardiac surgeries compared to a control group. For selection, two independent researchers have screened articles for the title and abstract for inclusion and exclusion criteria in order to eliminate duplicate documents to identify and select relevant topics. Then, full text of selected publications was evaluated by two authors independently. Any conflicts were resolved by consensus or the consult of a third author. Studies without adequate data or non‐human research were excluded, hence ultimately leading to a final total of 11 non‐randomized studies involved for analyses.

### 2.3 Quality Assessment

We used the Newcastle‐Ottawa Quality Assessment Scale (NOS) to evaluate the quality of the included studies [25]. This scale comprises eight elements that examine and evaluate the quality of relevant studies. The elements evaluate selection, comparability, and outcome according to the Ottawa checklist for cross‐sectional research. According to the final score on the NOS checklist, studies are categorized as very excellent quality (scores of 9–10), good quality (scores of 7–8), satisfactory quality (scores of 5–6), and inadequate quality (scores of 0–4).

### 2.4 Data Extraction

Extracted data for final analysis were as follows: study type, sample size, mean age of patients and controls, type of surgery, pre‐ and post‐ operative levels of S100B, and odds ratio of POCD. This study was approved by the Iranian National Committee for Ethics in Biomedical Sciences (Code of Ethics: IR. SBMU. RETECH. REC.1403.551).

### 2.5 Statistical Analysis

This meta‐analysis was performed to compare the concentration of S100B before and after surgery and its association with POCD patients compared to a control non‐POCD group using Stata version 18 (Stata Corp, College Station, TX, USA). Standardized mean difference (SMD) between patients and the control group were used as unit of analysis for the S100B. We used the cut‐off values set by Cohen for interpretation of small, medium, and large effect sizes (0.2, 0.5, and 0.8, respectively) [26]. Analyses were done using the random effects model. Heterogeneity was quantified by I^2^ statistics, for which values above 50% were considered to indicate moderate to high heterogeneity. We also conducted meta‐regression when there were adequate numbers of studies to investigate the Mini‐Mental State Exam (MMSE) as a potential effect modifier. Publication bias was assessed both visually by funnel plots and quantitatively by Egger's regression test.

### 2.6 Publication Bias

To evaluate the potential for publication bias among the included studies, we employed both visual and quantitative methods. First, we constructed a funnel plot of the studies reporting the association between S100B serum levels and POCD (Figure 5). In a funnel plot, the effect sizes of individual studies are plotted against their standard errors; a symmetric distribution suggests low risk of publication bias, whereas asymmetry may indicate potential bias due to unpublished studies or selective reporting

Second, we applied statistical tests to assess funnel plot asymmetry. Egger's regression test was performed to detect small‐study effects, with a *p*‐value < 0.05 indicating significant publication bias. Additionally, Begg's rank correlation test was used as a complementary method, assessing the correlation between effect estimates and their variances. In our analysis, Egger's test yielded a p‐value of 0.206, and Begg's test was not significant (*p* > 0.05), indicating minimal evidence of publication bias [27, 28].

## 3 Results

### 3.1 Study Selection

**Figure 1:**
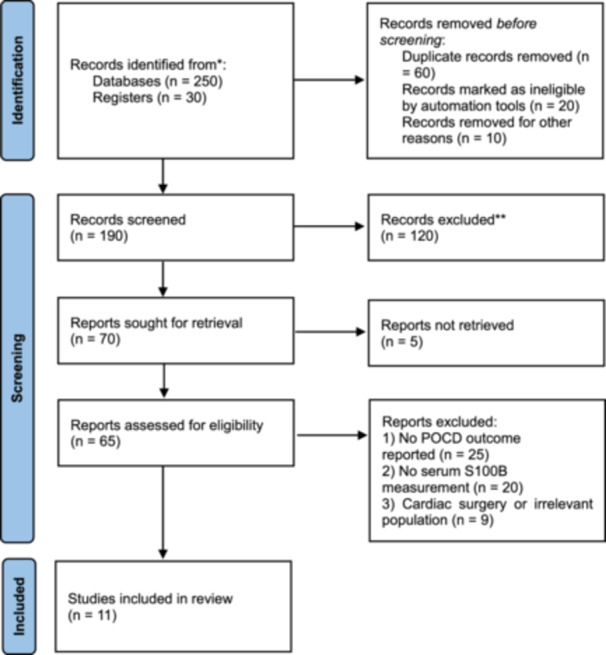
PRISMA flow diagram illustrating the study selection process for the meta‐analysis.

A total of 280 studies were identified through the database search, and after removing duplicates, 220 studies remained. Of the remaining papers, 150 were disregarded during the title and abstract screening process, and 59 were excluded after full‐text assessment. Finally, a total of 11 articles published from 2002 to 2022 were included in the meta‐analysis (Figure 1). The relatively small number of eligible studies highlights the limited but focused evidence specifically on non‐cardiac surgeries, emphasizing the need to consider differences in study populations and methodologies when interpreting pooled results.

### 3.2 Study Characteristics

The included studies were retrospective, and prospective observational as well as cohort, with follow‐up durations ranging from 6 days to a year. The quality assessment using the NOS revealed that the majority of studies were of high quality, with scores of 6 or higher (Table 1). Together, the studies involved 1207 subjects with individual study sample sizes ranging from 31 to 385 cases. Patients undergone vascular, trauma, urological, hip fracture, hip joint replacement, ear, nose and throat (ENT), lung or abdominal surgeries (Table 2). The diversity in surgery type and follow‐up duration could contribute to variability in S100B measurements and POCD incidence. Studies with shorter follow‐up may capture early postoperative cognitive changes, while longer follow‐ups may reflect persistent or delayed POCD. Differences in patient age and type of surgery may also underlie variations in observed S100B‐POCD associations.

**Table 1:** Quality assessment of the included studies on S100B levels and PCOD risk association.

Cohort studies	Representativeness of the exposed cohort	Selection of the non‐exposed cohort	Ascertainment of exposure	Demonstration that outcome of interest was not present at start of study	Comparability of cohorts on the basis of the design or analysis	Study controls for any additional factor	Assessment of outcome	Was follow‐up long enough for outcomes to occur (>10 years)	Adequacy of follow up of cohorts (loss‐to follow up < 20%)	Total score
Linsted (2002)	a	a	a	a	a	b	a	a	b	8
Beishuizen (2017)	a	a	a	a	a	b	a	a	b	8
Goettel (2017)	a	a	a	a	a	b	a	a	b	8
Ji (2016)	a	a	a	a	a	b	a	a	b	8
Ozturk (2020)	a	a	a	a	a	b	a	a	b	8
Alvin (2022)	a	a	a	a	a	b	a	a	b	8
Abdul Rahman (2022)	a	a	a	a	a	b	a	a	b	8
Yu (2017)	a	a	a	a	a	b	a	a	b	8
Munster (2009)	a	a	a	a	a	b	a	a	b	8
Molnar (2009)	a	a	a	a	a	b	a	a	b	8
Rasmussen (2000)	a	a	a	a	a	b	a	a	b	8

*Note:* Summary of Quality Assessment for Studies Investigating the Relationship Between S100B Levels and POCD Risk Using the Newcastle‐Ottawa Scale. According to the Newcastle‐Ottawa Scale (NOS) criteria.

**Table 2:** The Association Between S100B Levels and POCD.

Author [Year]	Country	Study design	Mean age (y)	Sample size	POCD	Non‐POCD	Type of Surgery	OR (95% CI)	Follow‐up time
Linsted (2002) [29]	Germany	Retrospective	64	117	48	69	Vascular, trauma, urological or abdominal surgery		6 days
Beishuizen (2017) (48)	Nederland	rct	83.9	385	169	216	Hip fracture surgery	1.56	12 Month
Goettel (2017) (49)	Switzerland	Observational cohort	73	82	38	44	Major noncardiac surgery	0.8	
Ji (2016) (50)	China	Prospective Observational	68.2	171	33	138	Hip joint replacement surgery	2.21	
Ozturk (2020) [23]	Turkey	Prospective Observational	63.2	82	24	58	RALRP	1.69	3 months
Alvin (2022) (51)	Indonesia	Prospective cohort	65	48	8	40	Major noncardiac surgery		
Abdul Rahman (2022) [30]	Indonesia	Prospective cohort	31.7	31	3	28	Ear, nose and throat (ENT) surgeries		
Yu (2017) (52)	China	Prospective cohort	51	71	28	43	Cytoreductive Surgery and Hyperthermic Intraperitoneal Chemotherapy (HIPEC)	1.25	7 days
Munster (2009) (53)	Nederland		83.9	120	62	58	Surgery after hip fracture	4.2	8 days
Molnar (2009) (54)	Hungary			35	9	26	Lung surgery		
Rasmussen (2000) (55)	Denmark		68	65	17	48	Abdominal surgery		3 Month

*Note:* Overview of Study Characteristics Examining the Association Between S100B Levels and POCD.

### 3.3 The Overall Association Between S100B Levels and Odds of POCD

**Figure 2:**
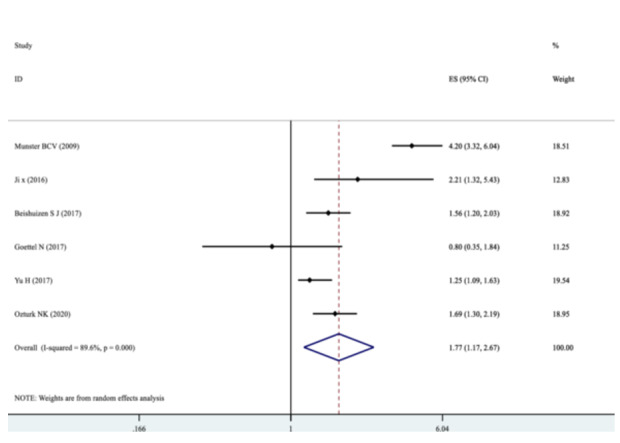
Forest plot showing the pooled SMD for the risk of POCD in patients undergoing non‐cardiac surgeries compared to controls.

**Figure 3:**
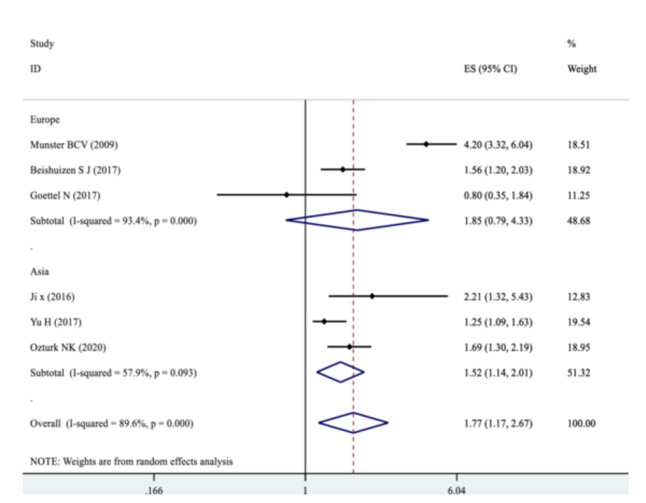
Forest plot showing the pooled smd for the risk of POCD in patients undergoing non‐cardiac surgeries based on the continent of origin (asia vs. europe).

**Figure 4:**
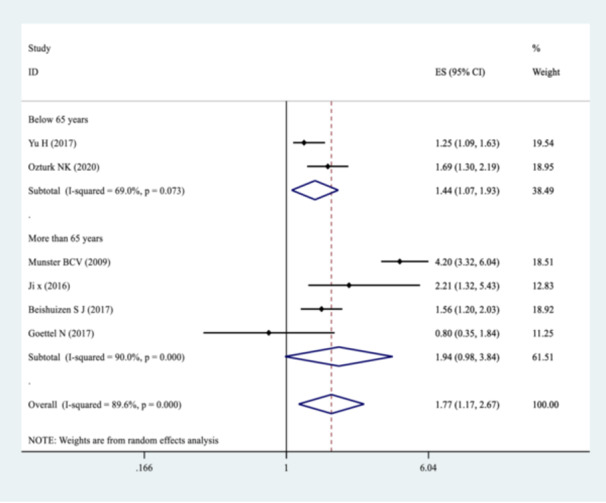
Forest plot showing the pooled smd for the risk of POCD in patients undergoing non‐cardiac Surgeries stratified by age groups (< 65 vs. ≥ 65 years).

**Figure 5:**
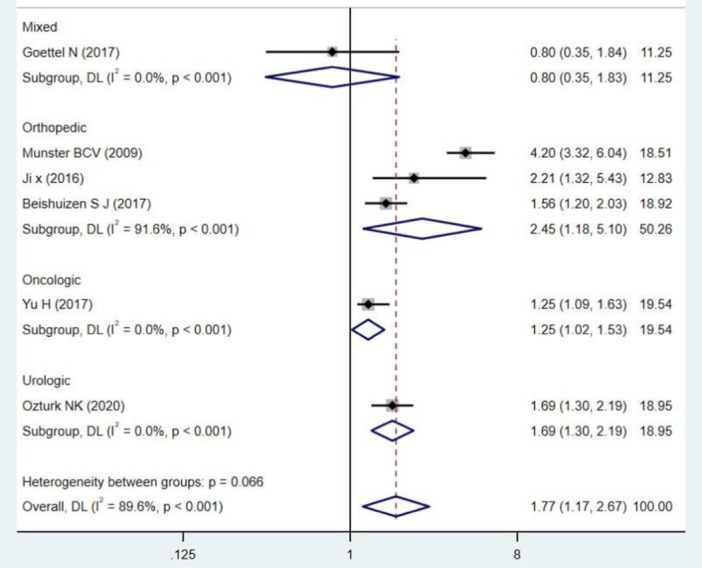
Forest plot of the risk of POCD in patients undergoing non‐cardiac surgeries according to the type of surgery.

Our pooled SMD analysis revealed that higher S100B levels were associated with a significant increase in the odds of POCD after non‐cardiac surgeries compared to non‐POCD control group, with the pooled SMD of 1.77 (95% CI: 1.17–2.67, *p* = 0.000, *I*² = 89.6%) (Figure 2). Subgroup analysis was performed for continent origin of the studies (Asia and Europe), as well as, patients age (below and above 65 years) (Table 3). The studies with Asian origin showed a significant association with POCD risk, with pooled SMD of 1.52 (95% CI: 1.14–2.01, *I*² = 57.9%, *p* = 0.093) (Figure 3). The pooled SMD for participants below 65 years was 1.44 (95% CI: 1.07–1.93, *I*² = 69.0%, *p* = 0.073), indicating a statistically significant effect (Figure 4). Subgroup analyses by the type of surgery demonstrated variable effect sizes. Only orthopedic surgeries showed a significant association with POCD (SMD = 2.45; 95% CI: 1.18–5.10), with high heterogeneity (*I*² = 91.6%). There were inadequate reports of other surgical categories such as oncologic, urologic surgeries, and mixed type of surgeries to be included in the meta‐analyses. The test for heterogeneity between surgical subgroups was not statistically significant (*p* = 0.066), suggesting no strong evidence of differential effects across surgery types (Figure 5).

**Table 3:** Subgroup Analysis for S100B Concentrations and Association S100B Levels and POCD.

	SMD or Pooled ES (95% CI)	*p*‐value	I2 (%)	P‐heterogeneity
**S100 levels Before surgery**				
Age				
Overall	0.00 (−0.12, 0.12)	0.990	0.0	0.384
<65 years	−0.02 (−0.12, 0.22)	0.991	0.0	
>65 years	0.03 (−0.12, 0.17)	0.984	0.0	
Continent				
Overall	0.00 (−0.12, 0.25)	0.990	0.0	0.854
Europe	−0.01 (−0.15, 0.14)	0.844	0.0	
Asia	0.02 (−0.21, 0.25)	0.972	0.0	
**S100 levels 30 min post‐op**				
Age				
Overall	0.14 (−0.10, 1.08)	0.700	0.0	—
< 65 years	0.14 (−0.10, 1.08)	0.700	0.0	—
> 65 years	—	—	—	—
Continent				
Overall	0.14 (−0.10, 0.39)	0.700	0.0	0.411
Europe	0.03 (−0.34, 0.39)	—	—	
Asia	0.23 (−0.09, 0.56)	0.688	0.0	
**S100 levels 24 h post‐op**				
Age				
Overall	0.10 (−0.07, 0.27)	0.959	0.0	0.625
< 65 years	0.14 (−0.10, 0.38)	0.770	0.0	
> 65 years	0.06 (−0.19, 0.30)	0.932	0.0	
Continent				
Overall	0.10 (−0.07, 0.27)	0.959	0.0	0.336
Europe	0.04 (−0.16, 0.25)	0.983	0.0	
Asia	0.23 (−0.08−0.53)	0.811	0.0	
**S100 levels 48 h post‐op**				
Age				
Overall	0.08 (−0.12, 0.28)	0.953	0.0	0.938
< 65 years	0.02 (−0.35, 0.39)	—	0.0	
> 65 years	0.10 (−0.16, 0.35)	0.756	0.0	
Continent				
Overall	0.08 (−0.12, 0.28)	0.953	0.0	0.705
Europe	0.05 (−0.18, 0.29)	0.909	0.0	
Asia	0.14 (−0.24, 0.52)	—	—	
**OR for the association of S100B levels and POCD**				
Age				
Overall	1.77 (1.17, 2.67)	0.000	89.6	0.000
< 65 years	1.44 (1.07, 1.93)	0.073	69.0	
> 65 years	1.94 (0.98, 3.84)	0.000	90.0	
Continent				
Overall	1.77 (1.17, 2.67)	0.000	89.6	0.000
Europe	1.85 (0.79, 4.33)	0.000	93.4	
Asia	1.52 (1.14, 2.01)	0.000	—	

*Note:* Results of Subgroup Analysis for S100B Levels and POCD Based on Age, Continent, and Postoperative Time Intervals.

Despite significant heterogeneity (*I*² = 89.6%), this overall effect suggests a consistent trend that elevated S100B may reflect neuroinflammatory responses linked to cognitive impairment. Heterogeneity likely stems from variations in patient age, type of surgery, timing of S100B measurement, and geographical differences in perioperative care.

### 3.4 Pre‐ and Post‐Surgical Levels of S100B

There were no significant pooled SMD of S100B levels in POCD subjects compared to non‐POCD individuals in pre‐surgical, and post‐surgical settings (30 min, 24 h, and 48 h) (Supplementary file). Although absolute S100B levels did not consistently differ at individual time points, the overall elevated odds of POCD suggest that relative elevations or patient‐specific responses, rather than absolute levels at single time points, may better indicate cognitive risk. Variations in measurement timing, surgery type, and patient factors likely contribute to these inconsistencies.

### 3.5 Publication Bias

**Figure 6:**
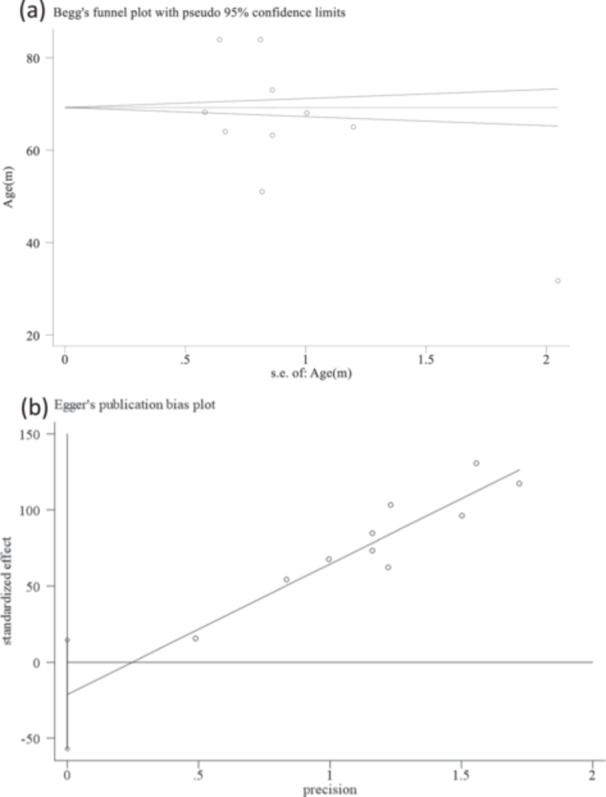
Publication bias assessment. (a) begg's funnel plot showing the symmetry of the studies included; (b) egger's plot assessing the statistical evidence of publication bias.

The Egger's test results for publication bias for studies evaluating the association between S100B and POCD risk presented no significant evidence of bias (Egger's test *p* value = 0.206). The Begg's funnel plot displayed an approximately symmetrical distribution of the studies included in the meta‐analysis, suggesting minimal evidence of significant publication bias (Figure 6). This indicates that studies with both positive and negative findings have likely been published. Importantly, most included studies were of moderate to high quality according to the Newcastle‐Ottawa Scale (NOS scores ≥ 6), suggesting that the observed results are less likely to be driven by low‐quality studies. However, variations in study populations, surgical types, and S100B measurement approaches could contribute to heterogeneity and may influence the robustness of the pooled estimates. Nonetheless, potential minor bias cannot be completely ruled out, and these factors should be considered when interpreting the findings.

## 4 Discussion

This meta‐analysis assessed the association between S100B levels and risk of POCD among patients undergone non‐cardiac surgeries. This study strengthens the evidence that non‐cardiac POCD is related to a peripheral inflammatory reaction [31]. Althouhg the pooled SMD of S100B concentrations did not show significant difference comparing POCD and non‐POCD patients, individuals with elevated S100B levels have 1.77 times increased odds for POCD (95% CI: 1.17–2.67).

The S‐100β protein has been known as a potential biomarker for neural damage and inflammation during brain injury [32, 33, 34]. Following central nervous system damage, glial cells become activated, leading to the release of S100B into the blood. Elevated serum concentrations of S100B may indicate glial damage or reactive astrogliosis, which could be associated with either beneficial or unwilling outcomes [14, 35]. Noteworthy, Serum levels of S100B protein may increase after both cardiac and non‐cardiac surgical procedures [36, 37, 38]. In surgical procedures not involving direct brain interaction, a rise in S100B may result from various clinical conditions that might lead to functional disturbance of blood‐brain barrier integrity leading to rupture or augmented permeability [39, 40]. Although controversies remain, previous studies demonstrated a correlation between the post‐operative cognitive dysfunction and S100β levels [15, 29]. A phase III randomized clinical trial evaluating the effect of dexamethasone on POCD also highlighted that serum levels of S100B have been considerably increased postoperatively [16]. Additionally, a compelling study also elucidated the correlation between POCD and elevated serum levels of S100B protein in patients undergoing Robotic‐Assisted Laparoscopic Radical Prostatectomy (RALRP) [18]. Evaluating 82 patients having RALRP, serum S100B protein levels were markedly elevated 30 min and 24 h post‐surgery in patients exhibiting POCD [18].

These findings indicate that the S100B protein may serve as a potential serum biochemical marker for central nervous system injury. However, our results showed there were no significant difference among S100B levels pre‐ and post‐surgery although considering 30 min, 24 h, and 48 h separately. High heterogeneity in our analysis proposes that the association between S100B levels and POCD can vary significantly in the presence of different cofounding variables. Linstedt et al. indicated that 30 min postoperative levels of S100 was higher in POCD group in abdominal and vascular surgeries but in urological surgery it had limited value [41], suggesting the effect of surgery type. Regarding inadequate data, we could not analyze the effects of surgery types in subgroups. Moreover, limited sample size in each study, varied demographic characteristics, diet, and anesthesia duration could also affect our findings [42]. Furthermore, the early timing of POCD assessment, particularly in the initial days post‐surgery, may be significantly affected by pharmacological interventions and also delirium [43]. Interestingly, according to subgroup analysis, studies conducted in Asian countries [30], demonstrated higher risk of POCD in relation to increased levels of S100B, when compared to European investigations. Although study findings might vary due to geographical variations due to different population health, surgical methodologies, and postoperative management, most of the studies reported the same results.

In older patients (over 60) undergoing non‐cardiac surgery, the International Study of Postoperative Cognitive Dysfunction (ISPOCD) reports a 25.8 incidence at week one and a 9.9% incidence at week three of POCD [44]. In contrast, a different study by Monk et al. discovered that among elderly patients who had non‐cardiac surgery, the incidence was 12.7% at 3 months and 41.4% at discharge [45]. Numerous factors, such as postoperative pain, hypoperfusion, thrombosis, inflammatory state, anesthetizing agents, etc., may play a role in the mechanisms of POCD, which are all affected by age [46]. Nonetheless, our results revealed that a statistically significant association between POCD and S100B levels in participants below 65 years old. In line with this finding, patients of younger age undergoing cardiopulmonary bypass, had higher pre‐operative cognitive scores; however, they had similar scores to older patients at the fourth day post‐operation [47]. Authors also showed higher levels of interleukin‐6 and CRP in youth, indicating that a perioperative inflammatory response may correlate with a decline in neurocognitive function, potentially more so in younger individuals compared to older ones. The included studies did not provide enough data based on early (minutes to days), and continued POCD in patients. Further large controlled trials should focus on the mechanism of inflammation and cognitive dysfunction after surgeries in different age groups to elucidate promising preventive approaches.

This meta‐analysis updates previous studies and also emphasizes new insights. An old meta‐analysis by Peng et al. was conducted with regard to the association of peripheral inflammatory markers, specifically S100B, with POCD [15]. It evidenced a significant association of high levels of such biomarkers with cognitive dysfunction, pointing to the importance of S100B in the elucidation of neuroinflammatory pathways leading to post‐surgical cognitive dysfunction. While Peng et al. included both cardiac and non‐cardiac surgeries, our study focused only on non‐cardiac surgeries; thus, more precise conclusions could be drawn about this subgroup. The present assessment also had more detailed subgroup analyses by age group (< 65 *vs.* ≥ 65 years) and geographic origin between Asia versus Europe, while Peng et al. only focused on biomarker concentrations.

Besides strengths, our study also has important limitations. The studies varied in size, with many of small samples, which reduces statistical power and limits generalizability. The significant heterogeneity across the included studies reduces the reliability of the pooled results. The study focused on immediate and short‐term POCD without exploring its long‐term implications, suggesting the necessity of large longitudinal studies. Despite different types of surgeries and cognition scales being included, we were unable to perform subgroup analysis by surgery type and cognition assessing scales regarding inadequate information. Moreover, the possible confounding variables, such as the type of anesthesia, surgical time, and patient comorbidities, were not well controlled. The results also may not be generalizable because of limited geographic representation and a focus on specific populations. Additionally, inconsistencies in the timing of S100B measurements post‐surgery make comparisons challenging. In addition, our analysis focused exclusively on non‐cardiac surgeries to avoid the physiological confounding factors inherent in cardiac procedures; therefore, the findings should not be generalized to cardiac surgery populations. Future large, longitudinal studies assessing inflammatory markers such as S100B are necessary to unravel the underlying mechanism and potential preventive strategy.

## 5 Conclusion

This meta‐analysis demonstrates that higher serum S100B levels are associated with increased odds of postoperative cognitive dysfunction (POCD) after non‐cardiac surgery (pooled OR = 1.77, 95% CI: 1.17–2.67). Subgroup analyses suggest that this association is influenced by age and geographical region, being more pronounced in patients under 65 years and in studies conducted in Asia. Although no significant differences were observed in pre‐ or post‐operative S100B concentrations overall, the longitudinal design of several studies indicates that S100B was measured before the onset of POCD, supporting its potential role as an early predictor.

## Author Contributions

**Saba Asefi and Kimia Jazi:** writing – review and editing, visualization, methodology, investigation, resources. **Dorsa Bahrami Zanjanbar, Pouya Omidi, and Azadeh Fateh:** writing – review and editing, writing – original draft, project administration, data curation, methodology, conceptualization. **Simin Sadeghi and Mohammadreza Hajiesmaeili:** funding acquisition, investigation, visualization, project administration, software, supervision.

## Disclosure

All authors have read and approved the final version of the manuscript and had full access to all of the data in this study and takes complete responsibility for the integrity of the data and the accuracy of the data analysis.

## Conflicts of Interest

The authors declare no conflicts of interest.

## Transparency Statement

The lead author Simin sadeghi affirms that this manuscript is an honest, accurate, and transparent account of the study being reported; that no important aspects of the study have been omitted; and that any discrepancies from the study as planned (and, if relevant, registered) have been explained.

## Data Availability

The data that support the findings of this study are available in the supporting material of this article.
